# Cysteinyl-glycine in the control of glutathione homeostasis in bovine lenses

**Published:** 2010-06-05

**Authors:** Gian Marco De Donatis, Roberta Moschini, Mario Cappiello, Antonella Del Corso, Umberto Mura

**Affiliations:** Department of Biology, Biochemistry Unit, University of Pisa, Pisa, Italy

## Abstract

**Purpose:**

To define a possible metabolic and/or signaling role for Cys-Gly in glutathione homeostasis in bovine eye lenses.

**Methods:**

Bovine lenses were cultured up to 24 h in a medium containing 0.5 mM reduced glutathione (GSH) under different conditions. The intracellular and the extracellular contents of thiol compounds were evaluated using a free zone capillary electrophoresis method.

**Results:**

Culture of lenses in the presence of GSH and the gamma-glutamyl transferase inhibitor serine-borate demonstrated a 1.5 fold increase in the level of extra-lenticular glutathione with respect to the initial value. Cys-Gly exogenously added impaired the extra-lenticular accumulation of glutathione. Both cysteine and gamma-Glu-Cys were ineffective in reducing extra-lenticular glutathione accumulation. In all conditions no differences in reduced and total intra-lenticular glutathione levels were observed.

**Conclusions:**

The impairment of Cys-Gly generation correlated with inhibition of gamma-glutamyl transferase by serine/borate, resulting in high extra-lenticular concentration of glutathione effluxed from the bovine lens. The possibility that Cys-Gly may intervene either in the replenishment processes for cysteine in the GSH biosynthetic step or in the function of the efflux GSH-transporters is considered.

## Introduction

Oxidative stress, generated by an imbalance between the action of prooxidant molecules and cell antioxidant systems, is one of the most relevant factors responsible for the impairment of normal cellular functions. The involvement of oxidative stress in the aging process and in the etiology of a variety of diseases is widely recognized [1,2]. Reduced glutathione (GSH), an abundant ubiquitous tripeptide, is involved in a variety of relevant biologic processes including detoxification of reactive metals and electrophilic molecules, cell cycle regulation, regulation of gene expression, apoptosis, membrane transport of both endogenous and exogenous molecules, and thermotolerance [3]. GSH is a relevant tool in the protection against oxidative damage, acting as a scavenger of reactive oxygen species (ROS) either directly [4,5] or as co-substrate of detoxifying enzymatic activities such as GSH peroxidase (E.C. 1.11.1.9). Its antioxidant effectiveness is elicited through the action of metabolically sustained (i.e., NADPH-dependent) glutathione reductase (E.C. 1.8.1.7). Moreover, the generally reversible process of thiol/disulfide exchange between oxidized glutathione (GSSG) and proteins, leading to the formation of glutathione-protein mixed disulfides, has been suggested as an enhancing mechanism of cellular antioxidant ability and as mechanism allowing the protection of protein sulfhydryls from irreversible oxidation [5-11]. New effects of GSH and a definition of its mechanisms of action under different physio-pathological conditions are extensively described in [12-15].

**Figure 1:**
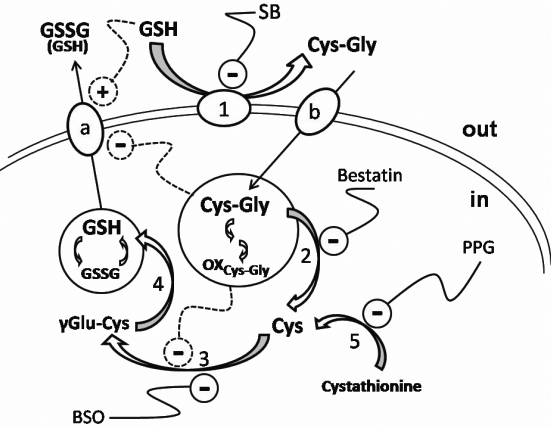
Schematic representation of γ-glutamyl cycle in the bovine lens epithelial-cortical layer. Numbers refer to enzymes involved in the cycle. 1: γ-glutamyltransferase; 2: leucine amino peptidase; 3, γ-glutamyl cysteine synthetase; 4: glutathione synthetase. “a” and “b” refer to glutathione and Cys-Gly transporters, respectively. The connection of the cycle with trans-sulfuration pathway (5: cystathionine γ-lyase) and the effect of possible modulators of the cycle are also reported. SB: serine /borate; PPG: propargylglycine; BSO: butionilsulfoximine. Inhibition or activation effects are indicated by “- “ or “+,” respectively. Dashed lines refer to the proposed effects on extralenticular GSSG accumulation exerted by exogenous GSH, Cys-Gly or/and its disulfide derivatives (OX_Cys-Gly_) as emerged from our results.

In the eye lens, where oxidative stress has been invoked as one of the factors involved in the etiology of cataract [16], GSH plays a major role in the maintenance and regulation of the thiol-redox status of the cell, counteracting the disruptive effect of oxidative stress on lens transparency and integrity. Apart from the possible decrease of GSH levels linked to the involvement of the tripeptide in detoxification phenomena, the cellular content of GSH is the result of two complementary processes constituting the so called γ-glutamyl cycle [4,17]: synthesis of GSH, from amino acidic precursors, and catabolism of GSH (see Figure 1). The latter catabolic component of the cycle is a multistep process beginning with extrusion of GSH through the cell membrane [4,17-20]. Indeed, GSH transporters in plasma membrane are key factors for the proper connection between GSH distribution and the GSH detoxification function [20]. Several reports suggest that extra-cellularly, glutathione occurs mainly in the GSSG form. Nevertheless, extra-cellular GSH has been reported to sustain distinct defense processes, such as the ocular surface and tear film defense mechanism [21,22] or in the aqueous humor as a first line antioxidant barrier against hydrogen peroxide [23]. Thus, glutathione efflux through the cell membrane in physiological conditions is unlikely to be a simple equilibrative process. Abnormal accumulation of external GSH may be indicative of pathological conditions mainly associated to γ-glutamyl transferase (γ-GT) deficiency, characterized by glutathionemie, glutathionuria, growth failure, shortened life span, and infertility [24,25]. Membrane bound ecto-enzyme γ-GT (E.C. 2.3.2.2) catalyzes the first step of GSH degradation, and is able to act on both reduced and oxidized glutathione [26]. One of the products of the transpeptidase reaction, the dipeptide Cys-Gly, can be recovered by the cell, possibly through the Pept2 transporter [27]. Cys-Gly is susceptible to hydrolysis by both membrane bound [28] and soluble dipeptidases [29] generating cysteine and glycine, which can be used for GSH intracellular re-synthesis. In the bovine lens, hydrolysis of Cys-Gly essentially occurs inside the lens, being the membrane bound dipeptidase poorly represented [29]. A peculiar feature of Cys-Gly is its pro-oxidant activity [5,30-32]. This feature has been indicated as the base for anti-apoptotic and proliferative cell signaling exerted by constitutive ROS deriving from γ-GT activity [32,33].

In this paper, we provide support for the hypothesis of a signaling and/or active role of Cys-Gly in the homeostasis of glutathione in bovine eye lenses during ex vivo culturing.

## Methods

### Materials

Cys-Gly, Gly-Gly, GSH, D,L-dithiothreitol (DTT), L-cysteine, L-serine, cystinyl-bis-glycine, γ-Glu-Cys, N-acetylcysteine, cystathionine, homocysteine, bestatin, propargylglycine (PPG), butionilsulfoximine (BSO), sodium tetra-borate deca-hydrate were purchased from Sigma Aldrich (Milan, Italy). Ninhydrin was from Merck (Darmstadt, Germany). Centricon 30 microconcentrators were from Millipore (Billerika, MA). ^3^[H]-Methylcholine chloride (1 Ci/ml) was from New England Nuclear (Boston, MA). Scintillation Liquid HiSafeII was purchased from GE Healthcare Europe, GmbH (München, Germany). Leucine amino peptidase (LAP) was purified from bovine lenses [29]. All other inorganic chemicals were of reagent grade.

### Lens incubation

Calf eyes were obtained from freshly slaughtered animals (age 1.5–3 years) at a local slaughterhouse and processed within 3 h of the animals’ death. Lenses were excised from bovine eyes using an anterior approach. Each lens (average weight±standard deviation: 1.70±0.15 g) was incubated at 37 °C with adhering vitreous (anterior side up) with gentle stirring in 40 ml of a phosphate buffered saline solution (2.7 mM KCl, 4.2 mM Na_2_HPO_4_, 1.5 mM KH_2_PO_4_) supplemented with 100 mg/ml streptomycin (standard medium). At appropriate times, 500 µl aliquots of the incubation medium were withdrawn, supplemented with 5 µl of 4 N HCl and frozen at −20 °C until use. At the end of incubation, each lens was checked for transparency; those few lenses that lost transparency during incubation were withdrawn from further study. After incubation, each lens was gently rolled onto filter paper to remove any adhering material, frozen and stored at −20 °C until analysis.

### Assessment of lens integrity

To assess the lens integrity, lenses were kept in the standard medium containing ^3^[H]-methylcholine chloride (6.25 nCi/ml) and the incorporation of the labeled compound was monitored. At different times lenses were collected, gently rolled onto filter paper, and homogenized in a Potter Elvehjem homogenizer with 6 ml of 10% trichloroacetic acid. After centrifugation at 10,000× g for 15 min at 4 °C, 400 µl aliquots of supernatant were added to 8 ml of scintillation liquid and radioactivity was measured in a Beckman LS5000CE liquid scintillator (Beckman, Fullerton, CA). The rate of choline incorporation after 28 h of lens culture [0.25±0.03 nCi/(h x g lens)] was greater than or equal to 80% of the incorporation rate measured during the initial 4 h of incubation.

### Measurement of thiol compound concentrations

The concentrations of thiol compounds, both in the lens culture medium and in the lens extracts, were measured by a free zone capillary electrophoresis (HPCE) method [34] with a Beckman P/ACE system 2000 using fused silica capillaries (50 cm×50 µm inner diameter) at a constant voltage of 30 kV, with 0.1 M tris-borate buffer, pH 8.5, as electrolyte. Each lens was homogenized at 4 °C in a Potter Elvehjem homogenizer with 5 ml of 50 mM KCl in 10 mM HCl; the homogenates were centrifuged at 10,000× g for 75 min at 4 °C and the supernatants were subjected to ultrafiltration through an Amicon Centricon 3. The ultrafiltrates were used for the determination of the concentrations of thiol compounds by HPCE. To determine the total glutathione (or other thiol compounds; i.e., thiol plus thiol disulfide plus protein-bound thiol), aliquots of the lens homogenates were brought to neutral pH by sodium hydroxide addition, treated with 5 mM DTT and kept for 2 h at room temperature before ultrafiltration through an Amicon Centricon 3.

### Lens dissection for enzyme distribution analysis

Frozen lenses were rapidly cut with a cork borer (1 cm inner diameter) into an anterior-posterior cylindrical shape, which was dissected in six transverse sections of about 1 mm. Each was weighed, homogenized by a Potter Elvehjem homogenizer in 1 ml of the appropriate buffer (i.e., 20 mM tris-sulfate buffer pH 7.6 for γ-glutamyl cysteine synthetase [γGCS] and glutathione synthetase [GSHS] or 10 mM sodium phosphate buffer pH 7.0 supplemented with 2 mM DTT for γ-glutamyl transferase [γGT] and LAP) and centrifuged at 10,000× g for 45 min at 4 °C. The supernatant was extensively dialyzed overnight against the same buffer before enzyme activity measurement.

### Enzyme activity assays

To assess the functional integrity of the lens, the level and distribution of γGT, γGCS, GSHS, and Cys-Gly hydrolase (i.e., LAP) were measured in the bovine lens. The activity of the enzymes was measured at 37 °C on each lens section as described below.

γGCS activity and GSHS activity were assayed by measuring the inorganic phosphate deriving from ATP cleavage in the proper assay mixture [35]. The γGCS assay mixture contained 100 mM tris-sulfate buffer pH 7.6, 15 mM L-glutamic acid, 5 mM L-cysteine, 2 mM ATP, and 50 mM MgSO_4_. The GSHS assay mixture contained 100 mM tris-sulfate buffer pH 7.6, 2.5 mM γ-glutamylcysteine, 30 mM glycine, 2 mM ATP, 5 mM MgSO_4_, and 100 mM KCl. For both γGCS and GSHS assays, phosphate concentration was evaluated by a reference curve obtained in the same assay conditions using inorganic phosphate as a standard. For both enzymes, one enzyme unit was defined as the amount of the enzyme that cleaves 1 µmole of ATP/min in standard assay conditions.

γ-GT activity was measured at 37 °C using GSH as substrate and Gly-Gly as a glutamate acceptor [36]. The assay mixture contained 100 mM tris-HCl buffer pH 8.5, 10 mM MgCl_2_, 0.2 mM MnCl_2_, 2 mM DTT, 20 mM Gly-Gly, 2.5 mM GSH and 40 mU/ml LAP. One enzyme unit was defined as the amount of the enzyme that catalyzes the conversion of 1 μmole of substrate/min in standard assay conditions.

LAP activity was measured at 37 °C by a colorimetric method using Cys-Gly as substrate [29]. Cysteine, produced from Cys-Gly hydrolysis, specifically reacts in acidic conditions with ninhydrin to form a complex showing a maximum absorbance at 560 nm (ε=34,800 M^−1^cm^−1^) [37]. The assay mixture contained 100 mM tris-HCl buffer pH 8.5, 0.5 mM MnCl_2_, 4 mM DTT, and 1 mM Cys-Gly. Cysteine concentration (from 0.05 to 0.4 mM) was evaluated by a reference curve obtained in the same assay conditions using cysteine as a standard. One enzyme unit was defined as the amount of enzyme that catalyzes the conversion of 1 μmole of substrate/min in standard assay conditions.

### Other methods

Protein concentration was estimated with the Coomassie blue binding assay [38] with BSA as the standard. Statistical analysis was performed with one-way ANOVA with Bonferroni’s posttest using GraphPad InStat version 3.0 GraphPad Software (San Diego, CA). If not otherwise specified, all the reported data are expressed as value±standard error of the mean.

## Results

### Level and distribution of enzymes in the bovine lens involved in the synthesis and breakdown of GSH

**Figure 2:**
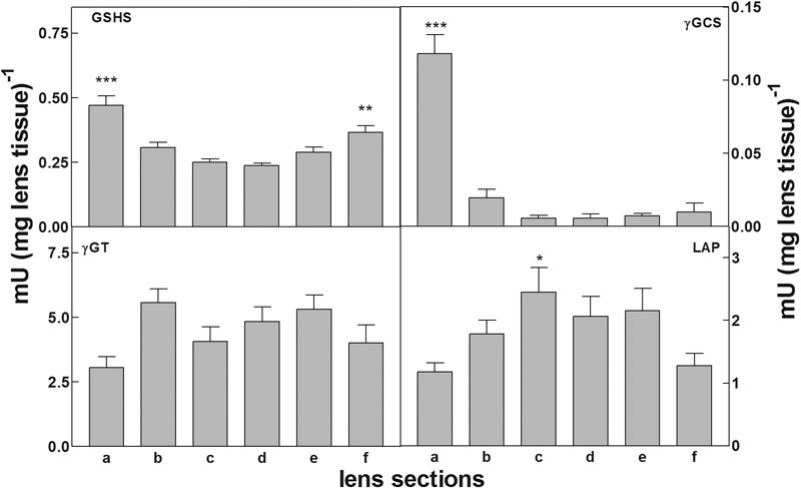
Lens enzymes distribution. Bovine lenses were dissected and different enzyme activities were assayed in each section as described in Methods. Slices “a” and “f” refer to the anterior and posterior sides, respectively. GSHS: glutathione synthetase; γ-GCS: γ-glutamyl cysteine synthetase; γGT: γ-glutamyl transferase; LAP: leucine amino peptidase. Values reported are the mean of three different experiments; the error bars represent the standard error of the mean. Significance was evaluated with respect to the slice displaying the lowest activity. (*): p<0.05; (**): p<0.01; (***): p<0.001.

The distribution and the level of the enzymatic activities responsible for the turnover of thiol intermediates of the γ-glutamyl cycle, were assessed in different lens regions (epithelium, cortex and nucleus) in lenses before and after 24 h incubation. In particular, the enzymes detected (see Methods) were γ-GT, γ-GCS, GSHS, and LAP. The results reported in Figure 2, referring to lenses at zero incubation time, revealed that while γ-GT, LAP and GSHS are highly represented in all lens regions, γ-GCS activity is present in the epithelium/outer cortex (section a), but is markedly reduced both in the inner cortical fibers and nucleus region. The same level and distribution pattern were observed in lenses after 24 h of incubation (data not shown). Thus the bovine lenses used in this investigation displayed the potential for GSH turnover, with the epithelium being the main active region for GSH synthesis.

### Incubation of lenses in the presence of GSH and the effect of serine/borate

**Figure 3:**
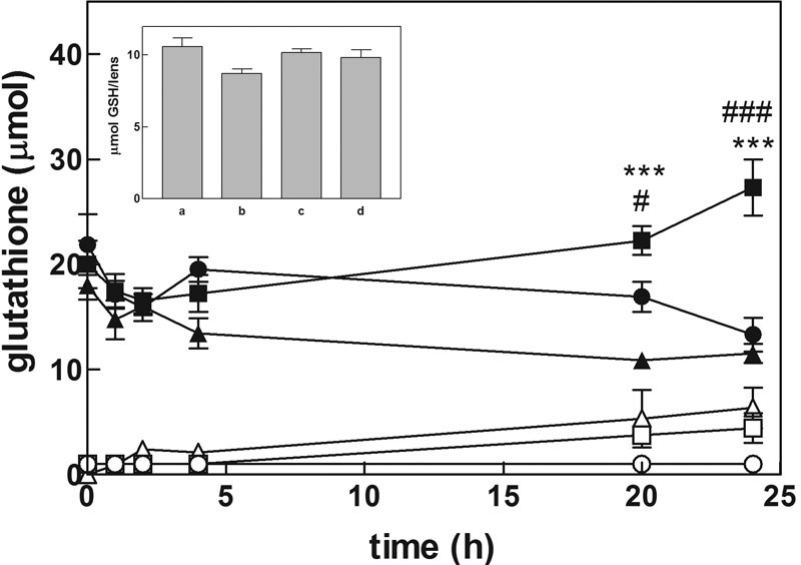
Changes in glutathione level during lens culture. Bovine lenses were incubated in a final volume of 40 ml in a GSH/Gly-Gly medium (see Methods) alone (circles), or in the presence of 10 mM serine/borate (squares) or 10 mM serine/borate and 0.5 mM Cys-Gly (triangles). Closed and open symbols refer to the extra-lenticular level of total glutathione and GSSG, respectively. Inset: bars refer to total glutathione intra-lenticular level measured after 24 h of incubation in the following conditions: a: standard medium; b: GSH/Gly-Gly medium; c: GSH/Gly-Gly/SB medium; d: GSH/Gly-Gly/SB medium supplemented with Cys-Gly. Statistical analysis was performed comparing the values measured under different conditions at the same time of incubation. (#): p<0.05 versus GSH/Gly-Gly medium, (###): p<0.001 versus GSH/Gly-Gly medium, (*): p<0.05 versus GSH/Gly-Gly medium supplemented with Cys-Gly, and (***):<0.001 versus GSH/Gly-Gly medium supplemented with Cys-Gly. Values are reported as the mean of at least five different experiments; the error bars represent the standard error of the mean.

**Figure 4:**
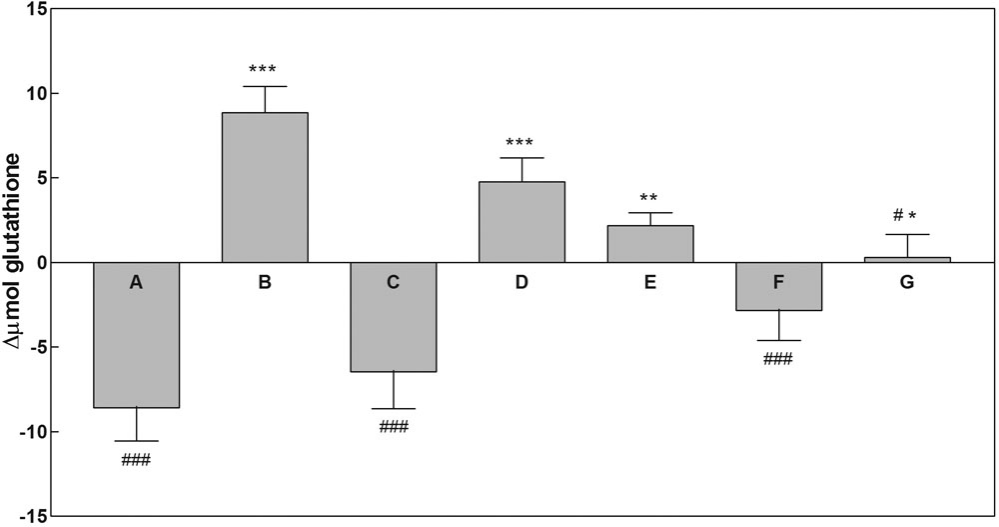
Changes of extra-lenticular level of glutathione under different lens culture conditions. Changes of extra-lenticular level of total glutathione are expressed as Δµmol glutathione, which refers to the difference between the value of extra-lenticular total glutathione (GSH plus GSSG expressed as GSH equivalents) measured at 24 h of incubation and that measured at zero time. Lens incubations were performed in a final volume of 40 ml in the following conditions: A: GSH/Gly-Gly medium; B: GSH/Gly-Gly/SB medium; C: GSH/Gly-Gly/SB medium supplemented with 0.5 mM Cys-Gly; D: GSH/Gly-Gly/SB medium supplemented with 0.5 mM cysteine; E: GSH/Gly-Gly/SB medium supplemented with 0.5 mM γ-Glu-Cys; F: GSH/Gly-Gly medium supplemented with 0.1 mM propargylglycine; G: GSH/Gly-Gly medium supplemented with 2 mM buthionine sulfoximine. See text for details. Values are reported as the mean of at least three different experiments; the error bars represent the standard error of the mean. Statistical analysis was performed by comparing Δµmol glutathione with respect to A (*) or B (#) conditions. (*, #): p<0.05; (**, ##): p<0.01; (***, ###): p<0.001.

Bovine lenses were incubated in standard medium supplemented with 0.5 mM GSH and 15 mM Gly-Gly (GSH/Gly-Gly medium). Glutathione levels were measured in the incubation medium over a 24 h period. At the end of incubation, each lens was processed as described in the Methods. The time course of extra-lenticular glutathione, as reported in Figure 3, showed a progressive decrease in total glutathione levels in the incubation medium (see also Figure 4, bar A). No significant differences were observed at the end of incubation in the intra-lenticular levels of total glutathione with respect to both lenses cultured in standard medium (Figure 3, inset) or untreated lenses (data not shown).

To evaluate the effect of γ-GT inhibition on glutathione levels, an equimolar mixture of serine and borate, which is known to competitively inhibit γ-GT activity, was used [39]. Thus lenses were incubated in the GSH/Gly-Gly medium in the presence of 10 mM serine/borate (GSH/Gly-Gly/SB medium). In these conditions the levels of extra-lenticular GSH equivalents increased by 1.5 fold relative to initial levels (Figure 3 and Figure 4, bar B). This increase was mainly due to accumulation of GSSG in the medium. Lenses assayed for total glutathione content at the end of incubation showed no significant differences between those lenses cultured in standard medium (Figure 3, inset).

### Effect of Cys-Gly on glutathione synthesis

**Figure 5:**
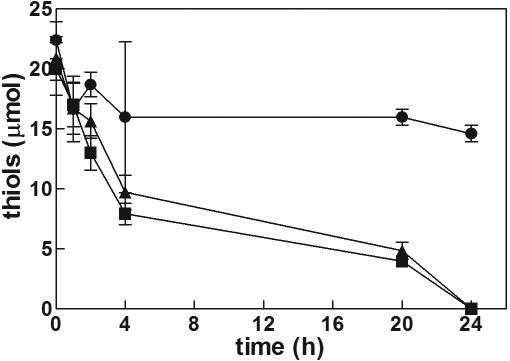
Extra-lenticular changes in cysteine, Cys-Gly and γ-Glu-Cys levels during lens culture. Bovine lenses were incubated in a final volume of 40 ml of GSH/Gly-Gly/SB medium supplemented with 0.5 mM of either cysteine (triangles), Cys-Gly (squares) or γ-Glu-Cys (circles) and at different times extra-lenticular thiol contents were evaluated (see Methods). Values are reported as the mean of at least three different experiments; the error bars represent the standard error of the mean.

When 0.5 mM Cys-Gly was supplemented to the GSH/Gly-Gly/SB medium, the extra-lenticular concentration of GSSG increased. Nevertheless, the extra-lenticular total GSH equivalents progressively decreased after 24 h, to a level comparable with that observed when lenses were incubated in GSH/Gly-Gly medium (Figure 3 and Figure 4, bar C). When lenses were assayed for glutathione content at the end of incubation, no difference was observed with respect to lenses cultured in standard medium (Figure 3, inset). Levels of exogenously added Cys-Gly progressively decreased to complete depletion at the end of incubation (Figure 5). These results suggest that Cys-Gly is able to prevent the synthesis of glutathione observed in the presence of serine/borate. The possibility that Cys-Gly acted by removing the inhibitory effect of serine/borate on γ-GT was ruled out, as 0.5 mM Cys-Gly was ineffective in interfering with the inhibitory action exerted by serine/borate on the γ-GT activity present in crude bovine lens extracts (data not shown).

### Effect of cysteine and γ-Glu-Cys on glutathione synthesis

The possibility that other thiol compounds, involved as Cys-Gly in the γ-glutamyl cycle, may influence the glutathione synthesis observed in the GSH/Gly-Gly/SB medium, was investigated. Lenses were incubated in the GSH/Gly-Gly/SB medium supplemented with 0.5 mM of either cysteine or γ-Glu-Cys. In the case of the cysteine supplemented medium, while the level of Cys progressively decreased in the incubation medium (Figure 5), the extra-lenticular level of glutathione showed a progressive increase at the end of incubation, up to a 1.30 fold with respect to the initial value (Figure 4, bar D). Similar results (i.e., an increase of extra-lenticular glutathione of 1.20 fold with respect to the initial value) were obtained in the presence of γ-Glu-Cys (Figure 4, bar E). In contrast to cysteine and Cys-Gly, which were completely consumed during incubation, γ-Glu-Cys is only partially consumed (Figure 5). No differences in total glutathione levels measured inside the lens were observed when cysteine or γ-Glu-Cys were present in the culture medium (9.70±1.48 µmol/lens and 9.90±2.16 µmol/lens, respectively) relative to incubation in standard medium (Figure 3, inset).

### Effect of compounds interfering with GSH synthesis

The possibility that the replenishment of cysteine necessary for glutathione synthesis could arise from the trans-sulfuration pathway was tested. For this purpose, PPG, an irreversible inhibitor of cystathionine γ-lyase, the enzyme that converts cystathionine to cysteine, was used [40]. Thus, lenses were incubated in GSH/Gly-Gly/SB medium supplemented with 0.1 mM PPG. Under these conditions, the extra-lenticular levels of glutathione at the end of incubation did not differ significantly from those observed in incubation in the GSH/Gly-Gly medium (Figure 4, bar F), suggesting that PPG almost completely prevented the synthesis promoted by serine/borate. No changes in extra-lenticular glutathione were observed when lenses were incubated in GSH/Gly-Gly/SB medium supplemented with 2 mM BSO (Figure 4, bar G), a known inhibitor of γ-GC-synthetase [41]. When lenses were analyzed for thiols content, no differences were observed in the total glutathione level with respect to lenses cultured in standard medium.

### Metabolic fate of Cys-Gly in bovine lenses

**Figure 6:**
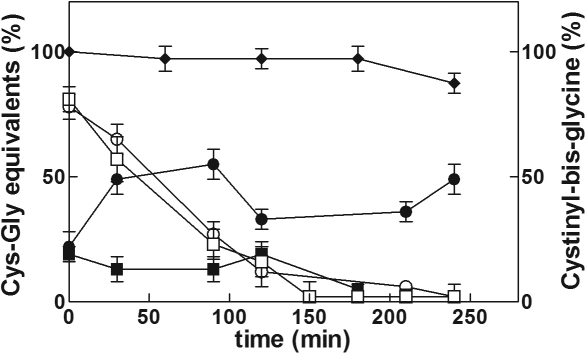
Extra-lenticular changes in Cys-Gly and cystinyl-bis-glycine levels during lens culture. Bovine lenses were incubated in 40 ml of standard medium containing 0.5 mM Cys-Gly alone (squares) or in the presence of 10 µM bestatin (circles); reduced and disulfide forms of the thiol (open and closed symbols, respectively) were measured and reported as % equivalents of the initial CysGly concentration. Diamonds refer to lens incubation performed in standard medium supplemented with 0.25 mM cystinyl-bis-glycine; the concentration of the disulfide in this incubation is reported as % of the initial value. Values are reported as the mean of at least three different experiments; the error bars represent the standard error of the mean.

Cys-Gly, when present alone in the standard medium (0.5 mM final concentration) (Figure 6), was consumed by the lens more rapidly than what was observed when incubated in GSH/Gly-Gly/SB medium. While a small amount of the homodisulfide cystinyl-bis-glycine could be detected in the medium, neither Cys-Gly nor cystinyl-bis-glycine were detected inside the lens. The level of total glutathione inside the lens remained constant during incubation (10.10±0.45 µmol/lens) and was not different with respect to the value measured in lenses cultured in standard medium. To evaluate the contribution of Cys-Gly hydrolizing enzymes to the metabolic fate of Cys-Gly, the lenses were pre-loaded with bestatin (10 µM bestatin in standard medium for 4 h), an effective inhibitor of dipeptidase activities [42,43], before addition of 0.5 mM Cys-Gly. In these conditions, while the extra-lenticular level of Cys-Gly was apparently unaffected by the dipeptidase inhibition treatment, the thiol compound appeared to consistently accumulate in the medium in its disulfide form (Figure 6). Intra-lenticular glutathione remained unchanged even as cystinyl-bis-glycine became detectable inside the lens (0.3±0.02 µmol/lens). Finally, as shown in Figure 6, cystinyl-bis-glycine exogenously added to the incubation medium (0.25 mM final concentration) was not taken up by the lens during incubation.

## Discussion

Our results highlight a potential connection between exogenous GSH and the intracellular control of GSH synthesis, mediated by Cys-Gly, a primary product of extra-lenticular GSH breakdown, that appears to play an active metabolic and/or signaling role (see Figure 1).

The reliability of the bovine lens model to monitor GSH homeostasis, was evaluated by both ensuring lens integrity as monitored by ^3^H-labeled choline incorporation, that showed the intake rate at the end of the lens incubation should not have been less than 80% of the initial value, as well as the function and stability of γ-glutamyl cycle enzymes. Levels and distribution of these lenticular enzymes related to GSH synthesis permitted GSH synthesis only in the epithelial and outer cortical regions (Figure 2). Similarly, the levels and distribution of γ-GT and LAP (Figure 2) enabled Cys-Gly metabolism in the lens, with LAP shown to be effective as a Cys-Gly hydrolyzing enzyme [29]. Immuno-histochemical detection of γ-GT confirmed its presence in the plasma membranes of both the lens epithelium and cortical fibers (Personal communication, Aldo Paolicchi). The lack of the rate-limiting enzyme of GSH biosynthesis γ-glutamyl cysteine synthetase in the lens nucleus may be key for the possible increase of Cys-Gly or cysteine levels produced by catabolic reactions and for reduced levels of GSH in older, nuclear lens regions, that may then favor conditions allowing oxidative damage to the crystallins observed during aging or cataract development [44,45].

As already mentioned, cellular extrusion of glutathione is part of the mechanism of cell recycling of the GSH tripeptide that is coordinated with intracellular redox activity and GSH synthesis (Figure 1), thus maintaining proper intracellular GSH levels. Maintenance of intracellular GSH homeostasis was observed even during the incubation of bovine lens in the presence of exogenous GSH (GSH/Gly-Gly medium) which did not change the intra-lenticular content of glutathione, even though we observed an overall decrease of glutathione levels in the medium by the end of the incubation period (Figure 3). Bovine lens incubated in the GSH/Gly-Gly medium supplemented with the γ-GT inhibitor serine/borate also demonstrated no significant change of intra-lenticular levels of glutathione (Figure 3, inset). However, the presence of serine/borate induces an increase of total glutathione level in the incubation medium, with a consistent accumulation of GSSG (up to 8.8±2.8 μmol GSH equivalents at 24 h; Figure 3 and Figure 4). From these data, the inhibition of γ-GT appears to drive synthesis of GSH, which is released by the lens and becomes detectable in the incubation medium. The preservation of the intra-lenticular homeostasis of glutathione supports our hypothesis that there is a strict control of the γ-glutamyl cycle, namely that the synthesis of the tripeptide is able to efficiently buffer the extrusion of glutathione. Extrusion of glutathione from bovine lens cultured in the absence of GSH and in the presence of serine/borate is rather low (0.63±0.23 μmol GSH equivalents at 24 h), which suggests an active role of the extra-lenticular GSH in the glutathione efflux from the lens.

While these results do not permit us to unequivocally relate the increase of GSH synthesis to the inhibition of γ-GT activity, the impairment of the GSH synthesis observed when Cys-Gly was supplemented to the GSH/Gly-Gly/SB medium supports the involvement of Cys-Gly in the negative control of glutathione synthesis/efflux from the lens.

The active removal of Cys-Gly from the incubation medium (Figure 6) may indicate the transport of this dipeptide into the lens. We found neither Cys-Gly, nor cysteine accumulated inside the lens either as reduced thiols, low molecular weight disulfides, or as thiol-protein mixed disulfides. The possibility that Cys-Gly may be metabolized through its hydrolysis to cysteine and glycine arises from our observations that after a preloading of the lenses with bestatin (a competitive slow-binding inhibitor of Cys-Gly hydrolase activities [42,43]) greater than 49% of Cys-Gly is found outside the lens as cystinyl-bis-glycine (Figure 6). Moreover, only under these conditions is a small amount of cystinyl-bis-glycine detectable inside the lens (0.30±0.02 μmol/lens). This result supports the notion that Cys-Gly is likely prone to oxidation within the lens and may in fact act as a “redox sponge,” inadvertently becoming a pro-oxidant. Interestingly, in our experiments cystinyl-bis-glycine exogenously added to the incubation medium remains outside the lens, likely as a consequence of the lack of degradation pathways and/or of the transport system (Figure 6). This observation suggests that cystinyl-bis-glycine accumulation observed in the presence of bestatin (Figure 6) may be dependent on the presence of external Cys-Gly. The requirement of the extra-lenticular reduced thiol to have extra-lenticular disulfide accumulation would correlate with the observed GSSG accumulation in the medium that occurred only in the presence of extra-lenticular GSH. It is likely that extracellular Cys-Gly would undergo hydrolytic breakdown, but its availability as a cysteine source does not seem to be a necessary requirement for GSH synthesis. In fact, we observed that glutathione synthesis is impaired in the presence of Cys-Gly when added to the lens culture medium, but is enhanced when the dipeptide generation is blocked through γ-GT inhibition with serine/borate. Cysteine is actively consumed during incubation without a concomitant increase of intra-lenticular thiol compounds (intra-lenticular cysteine level <0.6 µmol/lens). When cysteine rather than Cys-Gly is added to the lens culture medium, the GSH synthesis induced by exogenous GSH and serine/borate is not prevented (Figure 4). Similar results were obtained when γ-Glu-Cys was added to the GSH/Gly-Gly/SB medium, even though this compound was taken up by the lens to a reduced extent with respect to cysteine; approximately 25% of the initial value is consumed after 24 h of incubation (Figure 5). These results indicate that the ability of Cys-Gly to impair GSH synthesis induced by serine/borate does not depend on the intra-lenticular conversion of the dipeptide into cysteine, γ-Glu-Cys or their metabolites.

Although we have shown the specificity of action of Cys-Gly in impairing glutathione synthesis/efflux, these results do not address the specific target of Cys-Gly, or the final product of Cys-Gly metabolism. To address this question, an attempt to interfere with cysteine replenishment pathways was performed by supplementing the GSH/Gly-Gly/SB medium with propargylglycine, an inhibitor of cystathionine γ–lyase (E.C.4.4.1.1), the enzyme that catalyzes the conversion of cystathionine into cysteine. The impairment of the glutathione synthesis observed in these conditions suggests that trans-sulfuration pathway might contribute to the replenishment of GSH when the normal transmembrane glutathione recycle is impaired. Under similar conditions, buthionilsulfoximine, which is a known inhibitor of γ-GC-synthetase, is also able to prevent the extra-lenticular accumulation of glutathione (Figure 4). All the reported observations are outlined in Figure 1 that shows how the impairment of γ-GT, in the presence of extralenticular GSH, by limiting the availability of Cys-Gly, would remove the potential inhibitory effect of the dipeptide on the anabolic GSH pathway and/or GSH efflux mechanisms.

Thus, Cys-Gly either directly or through downstream metabolites may possibly interfere with cysteine homeostasis or with an aspect of GSH biosynthesis. Because Cys-Gly is susceptible to oxidation, it is conceivable that it might generate disulfide oxidation products such as cystinyl-bis-glycine or glutathione-Cys-Gly mixed disulfide in the lens, which may then affect GSH synthesis. This possibility might find support in the modest inhibitory effect (less than 10% inhibition) exerted by glutathione-Cys-Gly mixed disulfide that we observed in a preliminary kinetic investigation on partially purified γ-GC-synthetase (Unpublished data).

Another relevant key to further interpret the reported observations, may derive from the potential of Cys-Gly, a product of the membrane bound γ-GT, to intervene with the function of the GSH transporters. This hypothesis necessarily requires to envisage a mechanism enabling the simultaneous occurrence of these two events: i) the formation of Cys-Gly, which impairs the transporter(s) activity and ii) the presence of extra-lenticular GSH, which apparently drives the glutathione efflux.

In conclusion, the presented results show that, in cultured lens, the block of the γ-glutamyl cycle through γ-GT inhibition elicits extra-lenticular accumulation of glutathione, when exogenous GSH is present in the culture medium. The ability of Cys-Gly to abolish this effect suggests that this dipeptide exerts a negative control on glutathione synthesis/efflux in the lens. Factors and conditions underlying the mechanism of action of Cys-Gly are under active investigation.
